# Pleomorphic hyalinizing angiectatic tumor arising in the thigh: A case report

**DOI:** 10.3892/ol.2014.1883

**Published:** 2014-02-14

**Authors:** KAYO SUZUKI, TAKETOSHI YASUDA, TAKESHI HORI, TAKESHI OYA, KENTA WATANABE, MASAHIKO KANAMORI, TOMOATSU KIMURA

**Affiliations:** 1 Department of Orthopedic Surgery, University of Toyama, Toyama, Toyama 930-0194, Japan; 2 Department of Orthopedic Surgery, Iiyama Red Hospital, Iiyama, Nagano 89-2233, Japan; 3 Department of Pathology, Niigata Prefectural Central Hospital, Joetsu, Niigata 943-0192, Japan; 4 Department of Human Science 1, University of Toyama, Toyama, Toyama 930-0194, Japan

**Keywords:** pleomorphic hyalinizing angiectatic tumor, hemosiderotic fibrohistiocytic lipomatous lesion, imaging

## Abstract

Pleomorphic hyalinizing angiectatic tumors (PHATs) are rare non-metastasizing tumors of uncertain lineage. The current study presents a case of PHAT arising in the thigh of a 68-year-old female and examines the clinicopathological characteristics of the tumor. Magnetic resonance imaging (MRI) revealed an intramuscular mass located in the adductor longus. The tumor was surrounded by lipomatous tumor. Wide resectioning was performed for the internal tumor, whereas intralesional resectioning was performed for the external tumor. Histopathologically, the internal lesion was diagnosed as a PHAT and the external lesion was diagnosed as an hemosiderotic fibrolipomatous lesion (HFLL). No recurrence or metastases were identified during the 6-year follow-up period. As the adipose tissue surrounding the PHAT resembled a HFLL, therefore, the association between ‘early PHAT’ and HFLL is discussed. Although PHATs may represent low-grade sarcomas, HFLLs may be benign tumors.

## Introduction

Pleomorphic hyalinizing angiectatic tumors (PHATs) are rare neoplasms of the soft tissue that were first described by Smith *et al* in 1996 in a series of 14 cases (1). The tumor usually occurs within the subcutaneous tissue, particularly in the lower limbs of adults, and is histologically characterized by clusters of thin-walled ectatic vessels surrounded by hyalinized, fibrin and collagen material (2). Folpe and Weiss (3) described ‘early PHAT’ as an early stage or precursor lesion of classic PHAT that appears essentially identical to a hemosiderotic fibrolipomatous lesion (HFLL). The current study presents a case of PHAT arising in the thigh in which the adipose tissue surrounding the tumor mass resembled an HFLL. The patient was informed that data from the case would be submitted for publication, and written consent was subsequently obtained.

## Case report

A 68-year-old female presented with a solitary asymptomatic tumor of the left medial thigh that had been rapidly growing for ~2 months. There was no history of trauma and a physical examination revealed an elastic soft tumor measuring ~12×7 cm in size.

**Figure 1:**
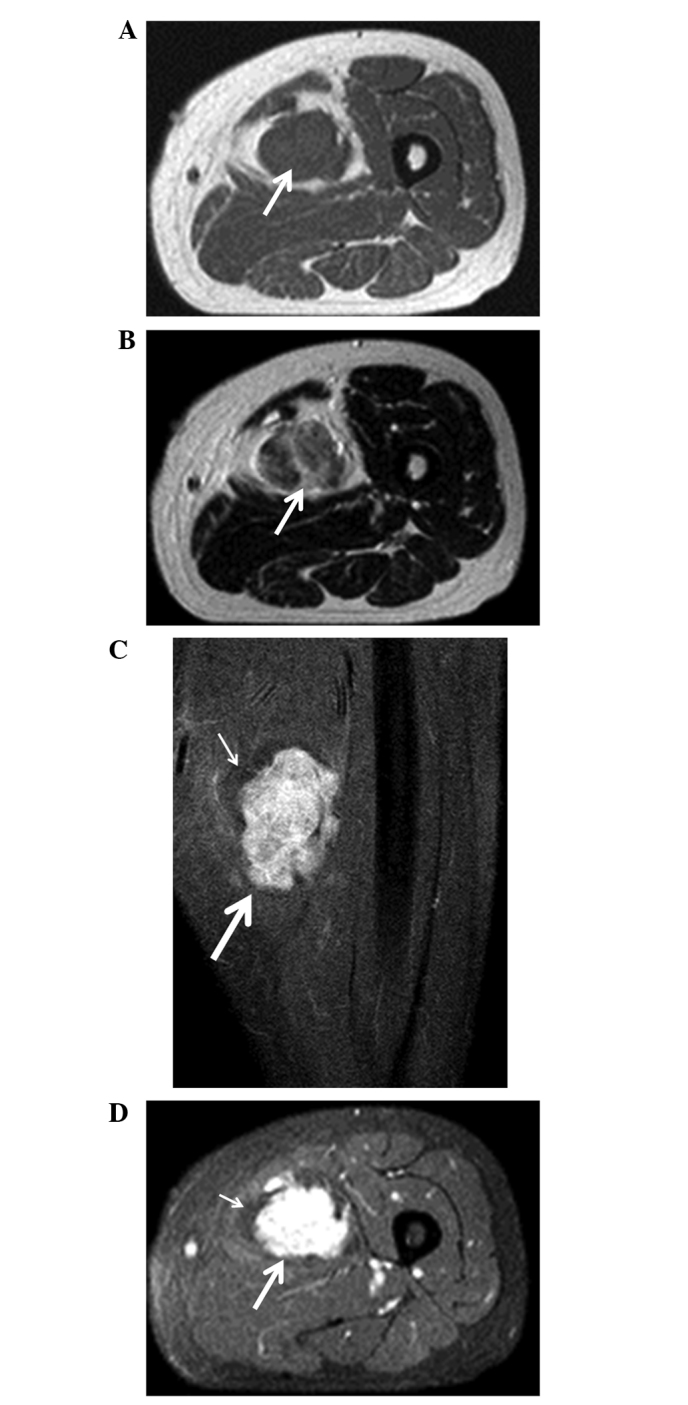
Magentic resonance imaging (MRI) findings on (A) T1- and (B) T2-weighted axial imaging revealing an intramuscular, low signal intensity mass located in the adductor longus (arrow). (C) Fat-suppressed gadolinium-enhanced frontal and (D) axial imagings revealing a multinodular, homogeneous, high signal intensity mass (large arrow). The surroundings of the mass are not enhanced (small arrow).

Radiography showed an expansion of the soft tissue on the medial aspect of the thigh, but no bony changes or calcifications. Magnetic resonance imaging (MRI) revealed that the tumor mass was isointense compared with the muscle on T1-weighted images (Fig. 1A). On T2-weighted images, the tumor was of heterogeneous high signal intensity (Fig. 1B). The tumor enhanced homogeneously following intravenous administration of gadolinium with gadolinium diethylenetriaminepentacetate (Gd-DTPA). The surrounding tissue was of high intensity on T1 and T2-weighted images, but was not enhanced following administration of Gd-DTPA (Fig. 1C and D). An angiogram showed that the tumor was fed by a femoral artery and was markedly stained. ^67^Gallium scintigraphy showed significantly abnormal uptake in the tumor. Furthermore, chest radiography and computed tomography found no evidence of lung metastasis, and the laboratory findings were normal.

**Figure 2:**
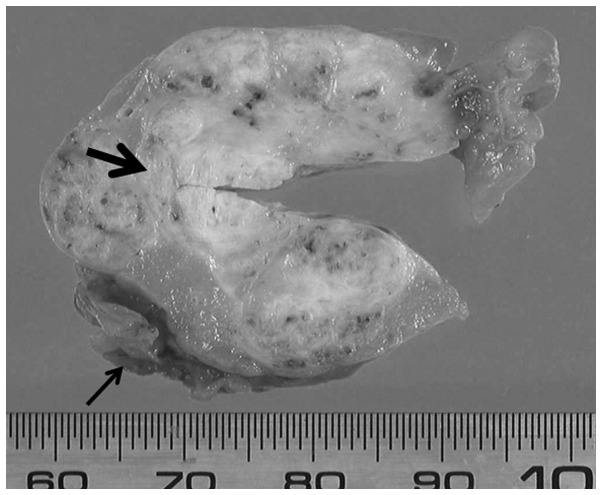
Gross appearance of the surgical specimen. The internal area is white-tan in color, hard and includes lipomatous tissue with a thin capsule (large arrow). The outer area is the lipomatous lesion (small arrow).

Based on a presumed diagnosis of a benign fibrous tumor or schwannoma, tumor excision was performed. The surgical findings concluded that the tumor was located in the femoral abductor muscle, with the femoral artery penetrating into the tumor. As a benign tumor was suspected according to the intraoperative frozen section examination, the majority of the mass was excised and the remaining portion was left attached to the artery. The gross appearance revealed two layers of structures: An internal lesion with a hard, white-tan colored tumor and an outer lesion composed of lipomatous tumor tissue (Fig. 2). A wide resection was performed for the internal lesion, whereas an intralesional resection was performed for the outer lesion. The tumor measured 10.5×4.5×3.5 cm and was lobular in appearance.

**Figure 3:**
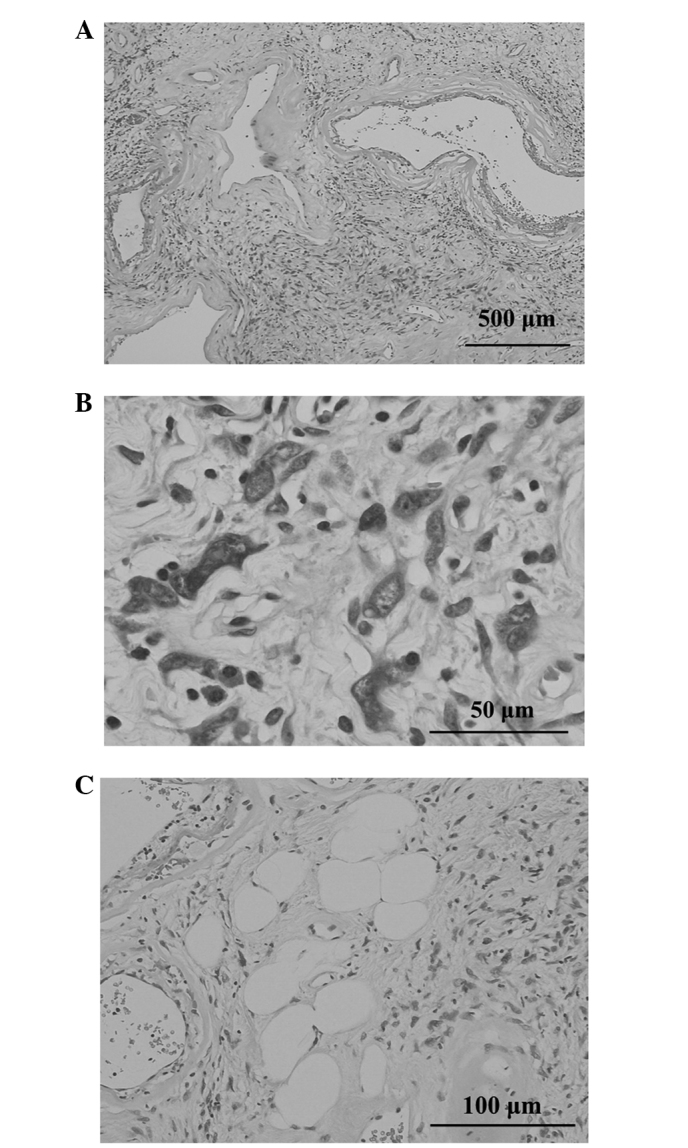
Histopathological findings of the surgical specimen. (A) Clusters of thin-walled ectatic blood vessels scattered throughout the tumor (hematoxylin and eosin stain). (B) Tumor cells showing prominent intranuclear cytoplasmic inclusions (hematoxylin and eosin stain). (C) Proliferation of bland spindle cells infiltrating mature adipose tissues (hematoxylin and eosin stain).

Histopathologically, the stroma revealed loose fascicles that were arranged in whorls or a haphazard, patternless fashion. This lesion contained a number of enlarged, thin-walled blood vessels with rims of a fibrinous or hyalinized material and organized thrombi and foci of hemosiderin deposition (Fig. 3A). The partially well-demarcated and lobular lesion was composed of a proliferation of spindle cells with hyperchromatic nuclei and nucleoplasmic bodies, as well as an eosinophilic or palely-stained cytoplasm occasionally admixed with pleomorphic cells (Fig. 3B). Mature adipocytes were present within the proliferation of spindle and pleomorphic cells (Fig. 3C).

Immunohistochemistry revealed a number of tumor cells were positive for vimentin, cluster of differentiation (CD)34, CD99 and B-cell lymphoma 2, whereas staining for S-100 protein, α-smooth muscle actin, desmin, AE1/AE3 and epithelial membrane antigen was negative. Mitotic figures were rare and the labeling index of Ki-67 was <3%. Based on these clinical and histological findings, a PHAT was diagnosed.

No recurrence or metastases were identified during the 6-year follow-up period.

## Discussion

PHATs are non-metastasizing soft-tissue tumors of uncertain lineage occurring within the superficial subcutaneous tissues and muscles (3,4). Presents in adults between the ages of 10 and 83 years (median age of 51 years), PHATs are more commonly observed in females than in males (4). The majority of affected patients present with slowly-growing, painless masses, most commonly involving the lower extremities. Rarer tumor sites include the arm, chest wall, axilla, popliteal fossa, buttocks, inguinal region, perineum, buccal mucosa and breast (2,4).

Due to its rarity, PHATs may be misdiagnosed as other soft-tissue lesions. Malignant fibrous histiocytoma and schwannoma should be considered within the differential diagnosis, as cellular pleomorphism and ectatic, hyalinized blood vessels with infiltration by variable chronic inflammatory cells is present in those types of tumors (5–7). The characteristics of a schwannoma, such as palisading of nuclei and the formation of Verocay bodies, are absent in a PHAT. Immunohistochemically, a PHAT is strongly positive for CD34, vascular endothelial growth factor and CD99, and negative for S-100 protein (1,7). In the present case, all findings were compatible with a diagnosis of a PHAT.

Currently, PHATs are a benign condition according to the World Heath Organization classification (2) and there have been no published studies of metastasis associated with PHAT to date. However, the local recurrence rate is 33–50% (1,3). Generally recurrences are not destructive in their growth (2); however, one previous study has described an aggressive recurrence that ultimately necessitated amputation (3). In rare cases, the tumors have recurred with the appearance of a sarcoma (3,8,9). These findings indicated that PHATs may be low-grade sarcomas.

A previous study reported an association between ‘early PHAT’ and HFLL (3). An HFLL is a reactive lesion that typically occurs in the foot or ankle region of middle-aged patients, and consists of an admixture of fat and moderately cellular fascicles of spindle cells. The lesion also shows vascular hyalinization and scattered pleomorphic cells (10). Folpe and Weiss (3) found a remarkable resemblance between ‘early PHAT’ and HFLL and suggested that an HFLL is a tumor related to a PHAT rather than a reactive lesion. In the present case, the outer lesion of the tumor may be an HFLL. As the femoral artery penetrated into the HFLL, a wide excision of the tumor, including the potential HFLL, was not possible. Although this portion of the lesion was left in place, the patient did not experience recurrence of the PHAT over the six-year follow-up period.

The published case studies of PHATs have primarily discussed the pathological findings of the disease. To the best of our knowledge, only one study has described the imaging characteristics of this tumor on MRI (11). In the present report, the association between the gross appearance and the imaging characteristics of the PHAT were analyzed. With regard to the gross appearance, the inside of the tumor was white-tan in color, hard and included lipomatous tissue, while the outside was made up of a lipomatous tumor. On MRI, the tumor was essentially isointense to neighboring muscles on the T1-weighted images. On the T2-weighted images, the signal intensity of the tumor was heterogeneously hyperintense. On the Gd-DTPA-enhanced images, the tumor mass was enhanced homogeneously. The existence of a large quantity of lipomatous tissues in the muscle indicated the possibility of an HFLL, and HFLLs are not enhanced following the intravenous administration of Gd-DTPA. Due to the histological overlap and the presence of areas that resemble HFLL and PHAT within the same tumor, certain studies have argued that HFLL is actually a precursor lesion to classic PHAT. Accordingly, the term ‘early PHAT’ has been used to distinguish it from classic PHAT, which exhibits more fully developed histological characteristics (3), although whether it should be considered an entity distinct from PHAT remains controversial (12,13).

Due to the considerable potential for local recurrence, surgical excision with a tumor-free margin is the preferred treatment for PHAT (4). Low-dose radiotherapy may be beneficial in cases of incomplete resection to avoid recurrence. Currently, there are no published reports of metastasis associated with a PHAT (1). The current study presented the case of a PHAT that was surrounded by an HFLL. PHATs may be low-grade malignant tumors; however, HFLLs are clearly benign lesions according to their histopathological characteristics. In the present study, removal was by a wide resection for the PHAT and by intralesional resection for the HFLL. The patient did not experience recurrence over the six-year follow-up period. This clinical course may result in a determination of the marginal range for this tumor. A wide resection is indicated for PHATs, but an intralesional resection may be used for HFLLs when they are in contact with important structures, such as an artery or nerve. Further studies are required to delineate the clinical course and long-term outcomes associated with this condition.
